# Design of the Nationwide Nursery School Survey on Child Health Throughout the Great East Japan Earthquake

**DOI:** 10.2188/jea.JE20150073

**Published:** 2016-02-05

**Authors:** Hiroko Matsubara, Mami Ishikuro, Masahiro Kikuya, Shoichi Chida, Mitsuaki Hosoya, Atsushi Ono, Noriko Kato, Susumu Yokoya, Toshiaki Tanaka, Tsuyoshi Isojima, Zentaro Yamagata, Soichiro Tanaka, Shinichi Kuriyama, Shigeo Kure

**Affiliations:** 1 Department of Disaster Public Health, International Research Institute of Disaster Science (IRIDeS), Tohoku University, Sendai, Japan; 1 東北大学災害科学国際研究所災害公衆衛生学分野; 2 Tohoku Medical Megabank Organization (ToMMo), Tohoku University, Sendai, Japan; 2 東北大学東北メディカルメガバンク機構; 3 Department of Molecular Epidemiology, Graduate School of Medicine, Tohoku University, Sendai, Japan; 3 東北大学大学院医学系研究科分子疫学分野; 4 Department of Pediatrics, School of Medicine, Iwate Medical University, Morioka, Japan; 4 岩手医科大学医学部小児科学講座; 5 Department of Pediatrics, School of Medicine, Fukushima Medical University, Fukushima, Japan; 5 福島県立医科大学医学部小児科学講座; 6 Area on Health Promotion Research, National Institute of Public Health, Wako, Saitama, Japan; 6 国立保健医療科学院地域保健システム研究分野; 7 Department of Medical Subspecialties, National Center for Child Health and Development, Tokyo, Japan; 7 国立成育医療研究センター生体防御系内科部; 8 Japanese Association for Human Auxology, Tokyo, Japan; 8 日本成長学会; 9 Department of Pediatrics, Graduate School of Medicine, The University of Tokyo, Tokyo, Japan; 9 東京大学大学院医学系研究科小児医学講座; 10 Department of Health Sciences, Graduate School Department of Interdisciplinary Research, University of Yamanashi, Chuo, Yamanashi, Japan; 10 山梨大学大学院総合研究部社会医学講座; 11 Department of Pediatrics, Graduate School of Medicine, Tohoku University, Sendai, Japan; 11 東北大学大学院医学系研究科小児病態学分野

**Keywords:** natural disaster, preschool children, physical development, children’s health, retrospective cohort, 東日本大震災, 未就学児, 身体発育, 小児保健, 後ろ向きコホート研究

## Abstract

**Background:**

The Great East Japan Earthquake inflicted severe damage on the Pacific coastal areas of northeast Japan. Although possible health impacts on aged or handicapped populations have been highlighted, little is known about how the serious disaster affected preschool children’s health. We conducted a nationwide nursery school survey to investigate preschool children’s physical development and health status throughout the disaster.

**Methods:**

The survey was conducted from September to December 2012. We mailed three kinds of questionnaires to nursery schools in all 47 prefectures in Japan. Questionnaire “A” addressed nursery school information, and questionnaires “B1” and “B2” addressed individuals’ data. Our targets were children who were born from April 2, 2004, to April 1, 2005 (those who did not experience the disaster during their preschool days) and children who were born from April 2, 2006, to April 1, 2007 (those who experienced the disaster during their preschool days). The questionnaire inquired about disaster experiences, anthropometric measurements, and presence of diseases.

**Results:**

In total, 3624 nursery schools from all 47 prefectures participated in the survey. We established two nationwide retrospective cohorts of preschool children; 53 747 children who were born from April 2, 2004, to April 1, 2005, and 69 004 children who were born from April 2, 2006, to April 1, 2007. Among the latter cohort, 1003 were reported to have specific personal experiences with the disaster.

**Conclusions:**

With the large dataset, we expect to yield comprehensive study results about preschool children’s physical development and health status throughout the disaster.

## Abstract (ja)

**背景：:**

東日本大震災（以下、大震災）は東北を中心とした東日本の太平洋沿岸地域に甚大な被害をもたらした。高齢者や障害者の健康への影響が懸念される一方で、震災が未就学児の健康へ及ぼす影響については殆ど知られていない。そこで、大震災前後における未就学児の身体発育と健康状態を調査するために全国保育所調査を実施した。

**方法：:**

調査は、2012年9月から12月に実施した。全国47都道府県の保育所に3種類の調査票を郵送した。調査票Ａでは保育所の状況を、調査票Ｂ１とＢ２では、園児の情報を収集した。対象は、2004年4月2日から2005年4月1日生まれの子ども‐保育所在園中に大震災を経験しなかった子ども‐、2006年4月2日から2007年4月1日生まれの子ども‐保育所在園中に大震災を経験した子ども‐である。調査項目は、被災経験、身体計測値、疾患の有無である。

**結果：:**

全国から3,624保育所が調査に参加した。その結果、2004年4月2日から2005年4月1日生まれの子ども53,747人と2006年4月2日から2007年4月1日生まれの子ども69,004人の2つの後ろ向きコホートを確立した。後者の中で、1,003名には、家屋の損壊、津波、避難所生活等の被災体験があった。

**結論：:**

得られた大規模なデータをもとに、大震災前後における未就学児の身体発育と健康状態に関する研究成果が期待される。

## INTRODUCTION

**Figure 1.**
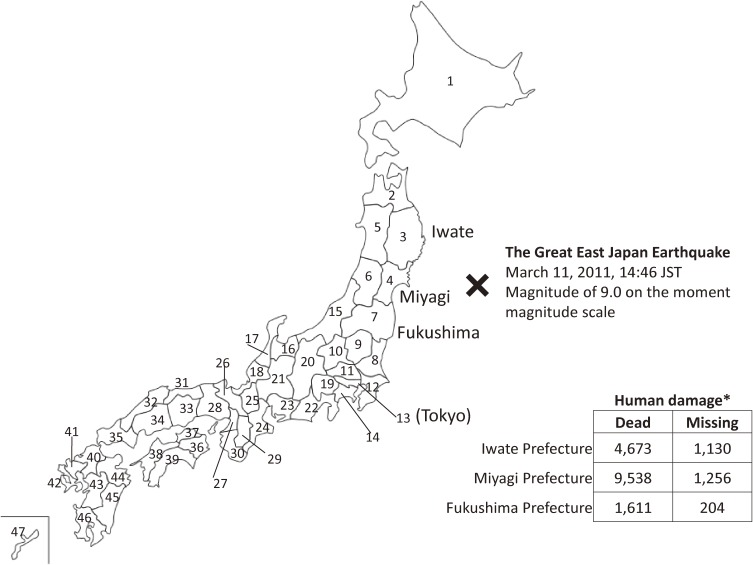
Geographic location affected by the Great East Japan Earthquake.The numbers on the map indicate prefecture codes corresponding to those in Table 1, Table 2, and Table 4.*The human damage number shows dead and missing persons in Iwate, Miyagi, and Fukushima Prefectures that were the most seriously affected by the Great East Japan Earthquake (The numbers are cited from Japan Meteorological Agency and National Police Agency).

The Great East Japan Earthquake, which occurred on March 11, 2011, was beyond our experience in modern Japanese history. The massive 9.0 magnitude earthquake was the largest quake ever recorded in Japan, and the following giant tsunami inflicted severe damage on the Pacific coastal areas of northeast Japan.^1^^–^^5^ The number of deaths and missing persons due to the disaster was 18 412 across Iwate, Miyagi, and Fukushima Prefectures (Figure 1).^6^ Furthermore, the earthquake caused a nuclear alert in the vicinity of the Fukushima Daiichi Nuclear Power Plant.^7^^–^^10^

Previous studies have reported health issues among the survivors and have focused attention on vulnerable populations, including the elderly, disabled, and hospitalized patients.^11^^–^^15^ Children are also vulnerable, but there has been little research documenting their health after the disaster.

In order to investigate the possible health impacts of the devastating natural disaster on preschool children, we conducted a nationwide nursery school survey. The survey should provide comprehensive and valuable epidemiological evidence of the impact of the disaster on preschool children, focusing on the differences in physical development before and after the disaster and assessing the extent to which experiencing the disaster, including environmental changes due to the disaster, may influence children’s health. This paper describes the design of the survey and the results of data collection.

## METHODS

### Survey design and population

We collected data on nursery school children not only from the most seriously affected areas of Iwate, Miyagi, and Fukushima Prefectures, but also from other areas across Japan. In the present survey, the prefectures indicate the location of the nursery schools that children were attending at the time of the survey. Prior to the survey, invitation letters were distributed to 23 711 authorized nursery schools,^16^ and 4266 (18%) nursery schools expressed interest in participating in the survey. From September to December 2012, we mailed three kinds of questionnaires to the 4266 nursery schools, and nursery teachers completed the questionnaires and mailed them back to the coordination office at Tohoku University.

**Figure 2.**
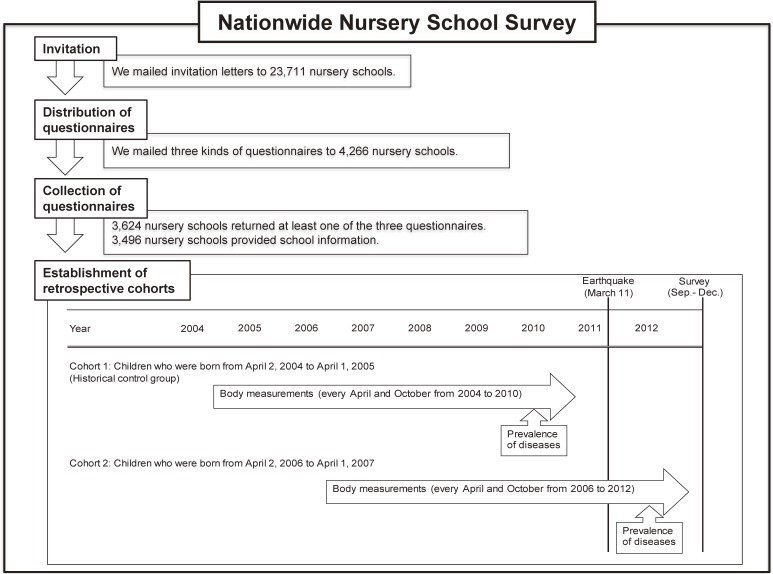
Flow of the Nationwide Nursery School Survey

The new school term in Japan starts on April 1, and a class consists of children who are born from April 2 to April 1 of the following year.^17^ We targeted children who were born in two classes: children who were born from April 2, 2004, to April 1, 2005, who were in the 5-year-old class of 2010 and did not experience the disaster during their preschool days; and children who were born from April 2, 2006, to April 1, 2007, who were in the 5-year-old class of 2012 and experienced the disaster during their preschool days (47 to 59 months of age when the disaster occurred). We defined the former group of children as a historical control group (Figure 2).

### Measurements

Questionnaire “A” addressed information on each nursery school: name of the nursery school, whether or not the nursery school was affected by the disaster, and the damage sustained in the disaster (collapse of the building, tsunami, fire, relocation of the nursery school, and others), if affected. Additionally, we asked for teachers’ subjective opinion through the question: “Do you think that experiencing the disaster influenced children’s development?” with an open-ended question about possible factors that might affect children’s development (eAppendix 1).

Questionnaires “B1” and “B2” addressed individual data on children who were born from April 2, 2004, to April 1, 2005 and those who were born from April 2, 2006, to April 1, 2007, respectively. Both anonymous questionnaires included questions about sex, year and month of birth, presence of diseases diagnosed by medical doctors (kidney disease, heart disease, atopic dermatitis, bronchial asthma, and others), history of moving in and moving out, and anthropometric measurements. According to the guidelines for childcare in nursery school, all nursery schools have to periodically perform physical measurements (generally every month) using a measurement procedure recommended by the Ministry of Health, Labour and Welfare.^18^ Considering the seasonal variation in growth, we retrospectively collected individuals’ height and weight measured in April and October for a maximum of 7 years. Additionally, we inquired about personal disaster experience with the following options: collapse of house, tsunami, fire, moving house, evacuation center, and death of a family member (eAppendix 2 and eAppendix 3).

### Ethical considerations

The survey protocol was approved by the institutional review board of Tohoku University. We collected only existing data, so we did not obtain informed consent from participants in either cohort. In accordance with the national Ethical Guidelines for Epidemiological Research, we disclosed information regarding the survey in two ways: we announced the conduct of the survey to parents using a poster displayed in each nursery school, and we disclosed the survey information, including the significance, objective, and methods of the survey, to the public on the website of Tohoku University’s School of Medicine at http://www.med.tohoku.ac.jp/public/ekigaku2013.html. Parents had the right to opt out.

## RESULTS

As shown in Table 1, nursery schools from all 47 prefectures participated in the survey. Of the nursery schools that agreed to participate in the survey, 3624 returned at least one of the three questionnaires. We acquired school information from 3495 nursery schools. We obtained individuals’ data for 54 558 children who were born from April 2, 2004, to April 1, 2005 (historical controls), and 69 702 children who were born from April 2, 2006, to April 1, 2007 (exposed children). As an initial data cleaning step, we excluded data on children who were born in a different year and those whose anthropometric measurements were not provided, leaving totals of 53 747 historical controls and 69 004 exposed children eligible for the initial dataset (Table 2).

**Table 1.** Proportion of nursery schools that participated in the survey

Prefecture		Number of nursery schools		Proportion

Code	Name	Target (*n* = 23 711)	Participation^b^ (*n* = 3624)	
1	Hokkaido	855	139	16%
2	Aomori	470	108	23%
3	Iwate^a^	359	81	23%
4	Miyagi^a^	346	132	38%
5	Akita	254	88	35%
6	Yamagata	241	42	17%
7	Fukushima^a^	317	97	31%
8	Ibaraki	489	53	11%
9	Tochigi	353	79	22%
10	Gunma	418	62	15%
11	Saitama	993	164	17%
12	Chiba	790	142	18%
13	Tokyo	1855	204	11%
14	Kanagawa	1142	120	11%
15	Niigata	709	156	22%
16	Toyama	303	62	20%
17	Ishikawa	361	50	14%
18	Fukui	272	40	15%
19	Yamanashi	231	37	16%
20	Nagano	586	60	10%
21	Gifu	425	42	10%
22	Shizuoka	510	98	19%
23	Aichi	1209	237	20%
24	Mie	477	77	16%
25	Shiga	208	21	10%
26	Kyoto	481	23	5%
27	Osaka	1236	95	8%
28	Hyogo	893	77	9%
29	Nara	192	25	13%
30	Wakayama	210	10	5%
31	Tottori	191	29	15%
32	Shimane	286	45	16%
33	Okayama	403	106	26%
34	Hiroshima	615	132	21%
35	Yamaguchi	310	53	17%
36	Tokushima	216	13	6%
37	Kagawa	209	41	20%
38	Ehime	320	49	15%
39	Kochi	258	44	17%
40	Fukuoka	905	144	16%
41	Saga	248	23	9%
42	Nagasaki	438	67	15%
43	Kumamoto	587	88	15%
44	Oita	280	37	13%
45	Miyazaki	394	66	17%
46	Kagoshima	473	48	10%
47	Okinawa	393	18	5%

^a^The three prefectures that were most severely affected by the earthquake include Iwate, Miyagi, and Fukushima Prefectures.

^b^We defined participation as returning at least one questionnaire from Questionnaire “A,” Questionnaire “B1,” and Questionnaire “B2.”

**Table 2.** Number of completed questionnaires returned from nursery schools

Prefecture		Questionnaire A: Questions regarding nursery school	Questionnaire B1: Questions for children born from April 2, 2004 to April 1, 2005	Questionnaire B2: Questions for children born from April 2, 2006 to April 1, 2007
		
Code	Name	(*n* = 3495)^b^	(*n* = 53 747)	(*n* = 69 004)
1	Hokkaido	137	1665	2087
2	Aomori	105	1135	1485
3	Iwate^a^	78	906	1248
4	Miyagi^a^	126	1804	2390
5	Akita	87	1463	1745
6	Yamagata	41	628	748
7	Fukushima^a^	97	1004	1557
8	Ibaraki	53	770	1137
9	Tochigi	77	1116	1519
10	Gunma	61	1180	1223
11	Saitama	155	2429	3235
12	Chiba	138	2488	3228
13	Tokyo	190	2573	4019
14	Kanagawa	118	2031	2551
15	Niigata	154	2020	3008
16	Toyama	61	1068	1092
17	Ishikawa	49	903	999
18	Fukui	39	408	580
19	Yamanashi	37	720	706
20	Nagano	55	1143	1292
21	Gifu	42	927	1096
22	Shizuoka	90	1886	2146
23	Aichi	231	5121	5588
24	Mie	73	1112	1437
25	Shiga	21	427	535
26	Kyoto	22	407	458
27	Osaka	91	1611	2273
28	Hyogo	72	1013	1464
29	Nara	25	334	500
30	Wakayama	9	178	201
31	Tottori	29	354	577
32	Shimane	45	482	699
33	Okayama	104	1778	2105
34	Hiroshima	125	2522	2982
35	Yamaguchi	51	534	853
36	Tokushima	12	157	156
37	Kagawa	40	462	753
38	Ehime	48	508	615
39	Kochi	43	653	763
40	Fukuoka	139	2571	3145
41	Saga	22	354	418
42	Nagasaki	65	647	770
43	Kumamoto	80	995	1336
44	Oita	36	311	467
45	Miyazaki	59	415	905
46	Kagoshima	46	452	774
47	Okinawa	17	82	139

^a^The three prefectures that were most severely affected by the earthquake include Iwate, Miyagi, and Fukushima Prefectures.

^b^Total number was not equal to 3624 as described in Table 1 because 129 nursery schools did not return Questionnaire “A.”

Table 3 briefly summarizes the characteristics of each cohort. The two cohorts were similar in distributions of sex, birth month, and presence of diseases diagnosed by medical doctors. Among children who experienced the disaster during their preschool days, 1003 (1.5%) were reported to have specific personal experiences with the disaster.

**Table 3.** Characteristics of nursery school children

	Children born from April 2, 2004 to April 1, 2005		Children born from April 2, 2006 to April 1, 2007		*P*
	
	*n*	%	*n*	%	
**Sex**					0.31
Boy	27 823	51.8%	35 536	51.5%	
Girl	25 449	47.3%	32 884	47.7%	
Missing	475	0.9%	584	0.8%	
**Birth month**					0.58
April	4556	8.5%	5657	8.2%	
May	4562	8.5%	5968	8.6%	
June	4404	8.2%	5733	8.3%	
July	4748	8.8%	5992	8.7%	
August	4676	8.7%	5946	8.6%	
September	4680	8.7%	6028	8.7%	
October	4405	8.2%	5693	8.3%	
November	4294	8.0%	5642	8.2%	
December	4361	8.1%	5682	8.2%	
January	4482	8.3%	5680	8.2%	
February	3771	7.0%	4801	7.0%	
March	4221	7.9%	5528	8.0%	
April (following year)	110	0.2%	114	0.2%	
Missing	477	0.9%	540	0.8%	
**Presence of diseases diagnosed by medical doctors**					0.28
No	44 380	82.6%	58 462	84.7%	
Yes	6064	11.3%	7832	11.4%	
Unknown	307	0.6%	342	0.5%	
Missing	2996	5.6%	2368	3.4%	
**Experience of the disaster**					
No	N/A		62 244	90.2%	
Yes	N/A		1003	1.5%	
Missing	N/A		5757	8.3%	
(Specific experience)					
Collapse of house			366		
Tsunami			224		
Fire			3		
Moving house			189		
Evacuation right			279		
Death of family member			31		

Differences in sex, birth month, and presence of diseases between two cohorts were tested by chi-square tests.

Table 4 presents the residential distribution of children with personal disaster experiences based on the location of the nursery schools that children were attending at the time of the survey. While 732 children (73.0%) were residing in Iwate, Miyagi, and Fukushima Prefectures, 271 (27.0%) were residing in various parts of the country other than the three affected prefectures.

**Table 4.** Residential distribution of children with personal disaster experiences

Prefecture		Disaster experience	

Code	Name	No (*n* = 62 244)	Yes (*n* = 1003)
1	Hokkaido	1911	4
2	Aomori	1372	14
3	Iwate^a^	1094	96
4	Miyagi^a^	1727	351
5	Akita	1650	8
6	Yamagata	665	31
7	Fukushima^a^	1116	285
8	Ibaraki	983	78
9	Tochigi	1395	6
10	Gunma	1101	5
11	Saitama	2942	11
12	Chiba	2987	41
13	Tokyo	3825	10
14	Kanagawa	2357	4
15	Niigata	2709	12
16	Toyama	984	0
17	Ishikawa	868	1
18	Fukui	551	0
19	Yamanashi	669	2
20	Nagano	1136	4
21	Gifu	985	0
22	Shizuoka	1966	3
23	Aichi	4974	7
24	Mie	1258	1
25	Shiga	489	1
26	Kyoto	402	0
27	Osaka	2063	2
28	Hyogo	1342	2
29	Nara	489	1
30	Wakayama	198	0
31	Tottori	552	1
32	Shimane	669	0
33	Okayama	1996	3
34	Hiroshima	2627	1
35	Yamaguchi	761	0
36	Tokushima	134	1
37	Kagawa	735	0
38	Ehime	571	1
39	Kochi	680	1
40	Fukuoka	2875	9
41	Saga	360	0
42	Nagasaki	702	0
43	Kumamoto	1229	3
44	Oita	442	1
45	Miyazaki	841	1
46	Kagoshima	729	1
47	Okinawa	133	0

	Three most affected prefectures^a^	3937	732
	Others	58 287	271

^a^The three prefectures that were most severely affected by the earthquake include Iwate, Miyagi, and Fukushima Prefectures.

## DISCUSSION

The present survey is the first nationwide survey to investigate how the Great East Japan Earthquake affected preschool children’s physical development and health status. The main strength of the present survey is the large amount of data we acquired. With the cooperation of 3624 nursery schools all over Japan, we established nationwide retrospective cohorts of 53 747 children who were born from April 1, 2004, to April 2, 2005, and 69 004 children who were born from April 1, 2006, to April 2, 2007. These cohorts represent 4.9% and 6.3% of the number of births in Japan during the same period, respectively.^19^

Preschool education in Japan is mainly provided either by nursery schools, which are governed by the Child Welfare Act and operate under the supervision of municipal governments,^16^^,^^20^^,^^21^ or by kindergartens, which are governed by the School Education Act^22^; a nursery school is a childcare and educational facility that cares for children ranging from newborn infants to preschool children, whereas a kindergarten offers early childhood education for children aged 3 to 5 years. Because nursery schools care for children for a longer period than kindergartens, we targeted nursery school children and obtained longitudinal data of physical measurements. Generalizability should be interpreted with caution. However, it has been reported that more than 40% of Japanese preschool children aged 3 years and older currently attend nursery schools and that the number of nursery school children has been increasing,^16^^,^^23^ so nursery school children may be sufficiently representative.

In addition, all nursery school teachers have paid close attention to children’s physical development by conducting periodic body measurements. They graduated from schools designated by the Ministry of Health, Labour and Welfare as educational institutions for nursery teachers, passed a national examination, and registered in the nursery teachers’ registry.^21^ Therefore, the anthropomorphic measurements obtained by such qualified teachers may be sufficiently reliable and accurate.

Ochi et al suggested that evaluations of the health impacts of disasters need baseline data from before the events.^11^ We therefore retrospectively collected nursery school children’s anthropometric measurements for a maximum of 14 times. Specifically, for children who experienced the disaster during their preschool days, we obtained their height and weight measured in April and October between 2006 and 2012, including 10 measurements before the disaster and four measurements after the disaster. Thus, the data reflect childhood physical development trajectories before and after the disaster.

We observed preschool children who had personal experiences with the disaster not only in Iwate, Miyagi, and Fukushima Prefectures, which were devastated by the disaster, but also in other areas all over Japan. Among 1003 children who were reported to have specific disaster experiences, 271 (27.0%) were residing outside of the affected prefectures. Because we conducted a nationwide survey, we collected valuable data, including data on children who might have moved from the affected areas.

In conclusion, by comprehensively examining the results from the present survey, we aim to provide valuable epidemiological evidence that may not only shed light on the impact of the Great East Japan Earthquake disaster on preschool children’s physical development and health, but may also provide specific suggestions for response to the next mega-disaster worldwide.

## ONLINE ONLY MATERIALS

eAppendix 1. Questionnaire A (Nursery school information).

eAppendix 2. Questionnaire B1 (Children who were born from April 2, 2004 to April 1, 2005).

eAppendix 3. Questionnaire B2 (Children who were born from April 2, 2006 to April 1, 2007).

Abstract in Japanese.
