# Supplementary material for: Design of the Nationwide Nursery School Survey on Child Health Throughout the Great East Japan Earthquake
Source: J Epidemiol. 2016 Feb 5;26(2):98–104. doi: 10.2188/jea.JE20150073 (PMC4728121; doi:10.2188/jea.JE20150073)
Supplement: eAppendix 1. [file je-26-098-s001.pdf]

eAppendix 1

Questionnaire A (Nursery school information)

Please answer the questions below in relation to your nursery school. Please circle the number or provide numbers or information in the underlined parts.

The date you filled out this questionnaire 2012 / month / day

The name of your nursery school ______________________

1. Was your nursery school affected by the Great East Japan Earthquake?

   1. Not affected 2. Affected

   → If affected, Circle one or more

   1. Collapse of the building 2. Tsunami 3. Fire
   4. Relocation of the nursery school

      Old address __________________________________

2. Do you think that experiencing the disaster impacted children's development? 1. Yes 2. No

   → If you answered yes, then what factors do you think influence children's development?

Thank you for your cooperation. We would like to request that you fill out Questionnaire B1 and B2.

Note: This version has not been verified for accuracy with the Japanese version.
