# Supplementary material for: Design of the Nationwide Nursery School Survey on Child Health Throughout the Great East Japan Earthquake
Source: J Epidemiol. 2016 Feb 5;26(2):98–104. doi: 10.2188/jea.JE20150073 (PMC4728121; doi:10.2188/jea.JE20150073)
Supplement: eAppendix 3. [file je-26-098-s003.pdf]

eAppendix 3

Questionnaire B2
Children who were born from April 2, 2006, to April 1, 2007
(Children in the 5-year-old class of 2012)
Nursery school personnel: please fill out one survey sheet per child.

For children born in fiscal year 2006

Please answer the following questions per child. Please circle the number or provide numbers or information in the underlined parts.

The date you filled out this questionnaire 2012 / month / day

- Sex 1. Boy 2. Girl
- Month of birth year / month
- Presence of diseases diagnosed by medical doctors (as of October 2012)
  1. Unclear 2. None 3. Yes
  → If you answered 'Yes' above, circle one or more below.
  a. Kidney disease b. Heart disease c. Atopic dermatitis
  d. Bronchial asthma e. Others (________________)
- History of moving-in and moving-out
  (Moving-in from _______ prefecture _______ nursery school in year / month)
  (Moving-out to _______ prefecture in year / month)
- Disaster experience and situation
  1. Did not experience the disaster 2. Experienced the disaster
  → If child experienced disaster, circle one or more below.
  a. Collapse of house b. Tsunami c. Fire d. Moving house
  b. Evacuation center f. Death of a family member
- Height and weight

	If you have records, please fill out.				Please fill out as many as possible.			
	2006 Apr	2006 Oct	2007 Apr	2007 Oct	2008 Apr	2008 Oct	2009 Apr	2009 Oct
Height, cm								
Weight, Kg								

	Please be sure to fill out. (If you do not have complete data, please provide data you have.)					
	2010 Apr	2010 Oct	2011 Apr	2011 Oct	2012 Apr	2012 Oct
Height, cm						
Weight, Kg						

In case of moving in or moving out, please answer as completely as possible.

Thank you for your cooperation.

Note: This version has not been verified for accuracy with the Japanese version.
