# Supplementary material for: Design of the Nationwide Nursery School Survey on Child Health Throughout the Great East Japan Earthquake
Source: J Epidemiol. 2016 Feb 5;26(2):98–104. doi: 10.2188/jea.JE20150073 (PMC4728121; doi:10.2188/jea.JE20150073)
Supplement: Abstract in Japanese. [file je-26-098-s004.pdf]

**東日本大震災前後における小児保健に関する全国保育所調査**

松原博子[1]，石黒真美[2,3]，菊谷昌浩[2,3]，千田勝一[4]，細矢光亮[5]，小野敦史[5]，加藤則子[6]，横谷進[7]，田中敏章[8]，磯島豪[9]，山縣然太郎[10]，田中総一郎[11]，栗山進一[1,2,3]，呉繁夫[2,11]
[1]東北大学災害科学国際研究所災害公衆衛生学分野、[2]東北大学東北メディカルメガバンク機構、[3]東北大学大学院医学系研究科分子疫学分野、[4]岩手医科大学医学部小児科学講座、[5]福島県立医科大学医学部小児科学講座、[6]国立保健医療科学院地域保健システム研究分野、[7]国立成育医療研究センター生体防御系内科部、[8]日本成長学会、[9]東京大学大学院医学系研究科小児医学講座、[10]山梨大学大学院総合研究部社会医学講座、[11]東北大学大学院医学系研究科小児病態学分野

**背景：**東日本大震災（以下、大震災）は東北を中心とした東日本の太平洋沿岸地域に甚大な被害をもたらした。高齢者や障害者の健康への影響が懸念される一方で、震災が未就学児の健康へ及ぼす影響については殆ど知られていない。そこで、大震災前後における未就学児の身体発育と健康状態を調査するために全国保育所調査を実施した。
**方法：**調査は、2012 年 9 月から 12 月に実施した。全国 47 都道府県の保育所に 3 種類の調査票を郵送した。調査票Ａでは保育所の状況を、調査票Ｂ１とＢ２では、園児の情報を収集した。対象は、2004 年 4 月 2 日から 2005 年 4 月 1 日生まれの子ども－保育所在園中に大震災を経験しなかった子ども－、2006 年 4 月 2 日から 2007 年 4 月 1 日生まれの子ども－保育所在園中に大震災を経験した子ども－である。調査項目は、被災経験、身体計測値、疾患の有無である。
**結果：**全国から 3,624 保育所が調査に参加した。その結果、2004 年 4 月 2 日から 2005 年 4 月 1 日生まれの子ども 53,747 人と 2006 年 4 月 2 日から 2007 年 4 月 1 日生まれの子ども 69,004 人の 2 つの後ろ向きコホートを確立した。後者の中で、1,003 名には、家屋の損壊、津波、避難所生活等の被災体験があった。
**結論：**得られた大規模なデータをもとに、大震災前後における未就学児の身体発育と健康状態に関する研究成果が期待される。

キーワード：東日本大震災、未就学児、身体発育、小児保健、後ろ向きコホート研究
